# Multi-scale ecological drivers shape the genome flexibility of soil-dwelling *Listeria monocytogenes*

**DOI:** 10.1093/ismeco/ycag093

**Published:** 2026-04-10

**Authors:** Ying-Xian Goh, F N U Hardeep, Hailong Zhang, Jingqiu Liao

**Affiliations:** Department of Civil and Environmental Engineering, Virginia Tech, Blacksburg, VA 24061, United States; Center for Emerging, Zoonotic, and Arthropod-Borne Pathogens, Virginia Tech, Blacksburg, VA 24061, United States; Global Change Center, Virginia Tech, Blacksburg, VA 24061, United States; Department of Computer Science, Virginia Tech, Blacksburg, VA 24061, United States; Department of Business Information Technology, Virginia Tech, Blacksburg, VA 24061, United States; Department of Civil and Environmental Engineering, Virginia Tech, Blacksburg, VA 24061, United States; Center for Emerging, Zoonotic, and Arthropod-Borne Pathogens, Virginia Tech, Blacksburg, VA 24061, United States; Global Change Center, Virginia Tech, Blacksburg, VA 24061, United States

**Keywords:** genomic variation, pangenome, environmental selection, bacterial communities, dispersal, *Listeria monocytogenes*

## Abstract

Understanding bacterial genome flexibility is key to predicting evolutionary trajectories and ecological interactions. Genome flexibility has been attributed to adaptive evolution, yet the underlying ecological drivers for bacterial pathogens persisting in natural environments remain poorly understood. *Listeria monocytogenes* (*Lm*), a foodborne pathogen prevalent in the environment, serves as an ideal model in this context. Through pangenome analysis of 177 *Lm* isolates representing three evolutionary lineages (I, II, and III) that we isolated from soils across the USA, we detected substantial genomic variation strongly associated with climatic factors, soil properties, and bacterial community composition, particularly the relative abundance of Nitrospirae, Planctomycetes, Acidobacteria, and Cyanobacteria. These factors were linked to many gene functions, particularly those involved in cell envelope synthesis, defense mechanisms, and replication, recombination, and repair. Among *Lm* lineages, distinct pangenome structure were observed. Lineage III exhibited a highly open pangenome, which was attributed to local adaptation to nutrient-limited conditions and strong dispersal limitation. In contrast, lineage I maintained a more conserved pangenome, likely due to frequent homogenizing dispersal across locations. Consistent with the dispersal patterns, lineage I showed an elevated risk for transmission along environmental-human pathways, evidenced by epidemiological links between three soil-derived and 17 clinical isolates. Collectively, this study suggests the pivotal roles of abiotic factors and bacterial communities in shaping genomic diversity of *Lm* and potentially other bacterial pathogens in soil. It also highlights significant differences in genome flexibility and transmission dynamics across lineages of the same pathogen species, underscoring the need for tailored source tracking strategies.

## Introduction

Genome flexibility, defined as the capacity of individual strains to acquire, lose, or reorganize genetic material, contributes to the pangenome variability observed in bacterial pathogens [1, 2]. Pangenome consists of core genes (i.e. genes present in all individuals) and accessory genes (i.e. genes not shared by all individuals) [3–5]. Some species, like *Escherichia coli*, have an open pangenome, where the number of genes increases substantially with each newly sequenced genome, while others, such as *Bacillus anthracis*, exhibit a closed pangenome, where the gene count remains stable despite the addition of more genomes [6–8]. While some argue that pangenome variability results from neutral evolution, our previous study and others suggest it is primarily caused by adaptation triggered by various abiotic and biotic pressures [8–10]. For example, antibiotic exposure was found to drive the acquisition of chloramphenicol and streptomycin resistance genes in *Vibrio cholerae* [11]*.* Also, mutualism between *Salmonella enterica* and *Methylorubrum extorquens* in co-culture has been shown to reduce the impact of loss-of-function mutations and selectively maintain nitrogen uptake genes in *S. enterica* compared to its monoculture [12]. While dispersal can promote the acquisition of novel genes by exposing pathogens to new environments [13, 14], it can also constrain overall genetic diversity through continuous movement of similar genetic material across populations [15–17]. Understanding how ecological properties influence these dynamics can shed light on the mechanisms and predictability of the evolution of bacterial pathogens under environmental changes and provide insights into transmission risks across environments. However, due to a lack of in-depth studies integrating genomics with paired abiotic and biotic environmental data, the ecological drivers of the genome flexibility of bacterial pathogens persisting in their natural reservoirs remain poorly understood.

*Listeria monocytogenes* (*Lm*) is characterized by high genomic diversification, ecological versatility, and public health relevance, making it an ideal model for studying the ecological mechanisms governing the genome evolution of bacterial pathogens. As a foodborne pathogen that causes rare but fatal listeriosis affecting vulnerable populations [18, 19], *Lm* can thrive and transmit across diverse habitats (e.g. soil, water, animals, food processing facilities, and hospital settings) [10, 19–22]. It has formed four distinct evolutionary lineages (designated as I, II, III, and IV) characterized by unique ecological roles [23, 24]. It is known that *Lm* genomes undergo adaptive evolution, particularly in food-associated environments [10, 25–28]. For example, *Lm* has developed stress survival islets (SSI) 1 and 2 to cope with environmental stress, with SSI-1 responsible for tolerating high salt and bile salt concentrations and low pH [25], and SSI-2 for tolerance of alkaline and oxidative stress conditions [26]. Our previous work on soil-dwelling *Listeria* demonstrated that pangenome variability is primarily driven by adaptive evolution [10]. However, the interactive roles of abiotic factors, microbiomes, and dispersal as key ecological drivers underlying the pangenome adaptive evolution remain underexplored.

To address this gap, we analysed the existing genomes of 177 *Lm* isolates representing lineages I, II, and III that we obtained from 1004 soil samples previously systematically collected from natural environments across the USA [10] and examined their relationships with 34 abiotic variables (geolocation, soil properties, climate, and surrounding land use) as well as bacterial community composition characterized by 16S rRNA gene sequencing. Using statistical and machine learning (ML) approaches, we identified evidence of strong effects of climate, soil properties, and bacterial community composition on gene richness across multiple functional categories, particularly cell envelope synthesis, defense mechanisms, and replication, recombination, and repair. We also identified distinct pangenome properties across *Lm* lineages contributed by local environmental adaptation and dispersal frequency. Informed by this observation, we identified a high risk of indirect or direct transmission from soil to humans in lineage I through comparative genomic analysis of soil and clinical isolates. Overall, this study provides insights into how selective pressures triggered by abiotic factors and bacterial communities as well as dispersal dynamics may contribute to the genome flexibility of *Lm* and potentially other bacterial pathogens in soil.

## Materials and methods

### Genomic data of soil *Lm* isolates, environmental data, and bacterial community data

Genome assemblies of 177 *Lm* isolates, classified into lineage I (12 isolates), lineage II (39 isolates), and lineage III (126 isolates), were obtained from soil samples collected by us from minimally disturbed natural environments across the USA and were previously published in our study investigating *Listeria* pangenome evolution through analyses of positive selection and homologous recombination [10]. These genomes represent the complete set of *Lm* isolates and their full geographic distribution reported therein [10]. In this study, they were subjected to secondary analyses, including correlations with environmental factors at the gene and functional levels, lineage-level pangenome comparisons, and comparisons with clinical isolates. Samples were collected using a systematic approach that achieved a relatively even spatial coverage as detailed in Liao *et al.* [10]. *Lm* lineage IV was not detected in soil in our nationwide survey [10], consistent with previous observations that *Lm* lineage IV is a rarely detected clade that has so far been found predominantly in ruminant animals (such as cattle or sheep), with very few reports, if any, from environmental reservoirs [23, 24, 29, 30]. Methods for *Listeria* DNA extraction, whole genome sequencing, and sequencing data processing were detailed in Liao *et al.* [10]. Genome assembly quality control metrics were provided in the Supplementary Information. Orthologous genes were previously identified using MMseqs2 [31] and annotated for Clusters of Orthologous Group (COG) functions using eggnog-mapper2 [32]. In this study, core genes are defined as those present in exactly all genomes analysed for a given bacterial population (e.g. a specific *Lm* lineage), while accessory genes are those not shared by all genomes [6–8]. Pangenome refers to the complete set of gene families identified across all genomes of a given population (i.e. the sum of core and accessory genes) [6, 33].

Environmental data analysed in this study contained 34 variables, including three geolocation variables (longitude, latitude, elevation), 17 soil physicochemical property variables (moisture, total nitrogen [TN], total carbon [TC], pH, organic matter [OM], aluminum [Al], calcium [Ca], copper [Cu], iron [Fe], potassium [K], magnesium [Mg], manganese [Mn], molybdenum [Mo], sodium [Na], phosphorus [P], sulfur [S], and zinc [Zn]), four climate variables (precipitation, wind speed, maximum and minimum temperatures), and 10 land use variables (proportions of open water, barren, forest, shrubland, grassland, cropland, pasture, wetland, and developed [Dev] open space with >20% and <20% impervious [IMP] cover in surrounding area). These environmental data were published in Liao *et al.* [10].

Bacterial community data, with detailed methods for DNA extraction, 16S rRNA gene amplicon sequencing, raw sequence read processing, and operational taxonomic units (OTUs) identification, taxonomy classification, and diversity calculation (including weighted UniFrac distances), were published in our previous study comparing *Listeria* populations from natural and food-associated environments [20], and underwent secondary analysis, including variation partitioning analysis (VPA), correlation analysis, redundancy analysis (RDA), and ML, to examine associations with *Lm* genome content in this study.

### Gene richness analysis

Gene richness, defined as the total number of unique orthologous genes per genome, is a well-established metric commonly used to quantify genomic variation and to assess how environmental, ecological, and evolutionary factors shape genome composition [34–36]. To identify key abiotic and biotic factors associated with gene richness, VPA and correlation analysis was conducted. VPA was employed to assess the contributions of (i) geolocation, soil properties, climate, and surrounding land use, and (ii) abiotic factors (i.e. all four groups in (i) combined) and biotic factors (i.e. the relative abundance of bacterial phyla) to the variation of gene richness overall and for COGs at both species and lineage levels. VPA uses multiple linear regression to partition the total variation into fractions uniquely explained by each predictor group and those shared among groups. VPA was computed and the adjusted *R^2^* values were visualized in a Venn diagram using the vegan package 2.6-4 in R. To assess the significance of the explained variation, a permutation test, detailed in the Supplementary Information, was performed. Spearman’s correlation was further conducted to quantify the associations between individual abiotic and biotic factors and overall gene richness and for each COG. To account for multiple testing, *P*-values were adjusted using the Benjamini–Hochberg false discovery rate (BH-FDR) method.

### Redundancy analysis, machine learning models, and identification of genes associated with abiotic and/or biotic factors

To identify key abiotic and biotic factors associated with accessory genomic variation, RDA and ML-based modelling were employed. RDA was selected over canonical correspondence analysis based on detrended correspondence analysis [37] to identify relationships between abiotic/biotic variables selected by L1 regularization (Lasso) [38] and presence/absence matrix of accessory genes (methods detailed in the Supplementary Information). RDA is a constrained ordination method that uses linear regression and principal component analysis to quantify the variation in accessory genomes explained by the predictors. To predict the RDA ordination axes from abiotic and biotic factors separately, we implemented a ML-based framework using Python 3.6.8 reported in our previous study [39]. Briefly, 34 abiotic environmental variables and 27 bacterial phyla were used as predictors across 177 samples. The data were preprocessed and split into training (80%) and testing (20%) sets. Various regression models were trained and evaluated with optimized hyperparameters using five-fold cross-validation (see Supplementary Table 1). To identify the best-performing model, we compared various algorithms, including regressors based on random forests, neural networks, gradient boosting, support vector machines, and *k*-nearest neighbors. The model with the highest *R^2^* score was then fine-tuned to achieve optimal performance. The importance of the features to the prediction was quantified using Shapley Additive exPlanations (SHAP) [40].

Two-sided Mann–Whitney (MW) *U* tests followed by BH-FDR adjustment were employed to compare abiotic and biotic factors between genomes with and without a given accessory gene. Functional enrichment analysis was conducted using a binomial distribution model described in Liao *et al.* [20] to identify COGs significantly enriched among genes associated with abiotic and/or biotic factors.

### Environmental characteristics of *Lm* lineage habitats and distance–decay relationship

A previously published spatial distribution map of 177 *Lm* genomes (**Extended Data Fig. 5** in Liao *et al*. [10]) was adapted using the Basemap Matplotlib Toolkit 1.2.1 in Python 3.6.8. To identify significant differences in abiotic and biotic factors among *Lm* lineages, Kruskal–Wallis (KW) tests with BH-FDR correction were performed, followed by pairwise comparisons using two-sided MW *U* tests with BH-FDR correction. Multidimensional scaling (MDS) analysis was performed using Euclidean distance for abiotic factors and weighted UniFrac distance for OTUs followed by a PERMANOVA test to identify differences in abiotic conditions and microbial community composition among lineages. To infer dispersal limitation, distance–decay relationships were assessed by regressing average nucleotide identity (ANI) against geographical distance for each lineage.

### Pangenome features of *Lm* lineages

To predict the pangenome size (i.e. the total number of genes) given 100 genomes for each *Lm* lineage, we applied the power law function *cN^γ^*, calculated in **Extended Data Fig. 7** in Liao *et al*. [10]. In this function, *c* is the size of the core genome size, *N* is the number of genomes, and *γ* is a scaling exponent fitted to phylogroup-specific pangenome rarefaction curves [10]. Pangenome openness is defined by *γ*, with values closer to 0 indicating a more closed pangenome [10]. The specific functions used were n_pan_ = 2723 *N*^0.093^ for lineage I, n_pan_ = 2681 *N*^0.122^ for lineage II, and n_pan_ = 2490 *N*^0.159^ for lineage III [10]. Notably, *γ* does not vary monotonically with sample size *N*. For example, *Lm* lineage III had more isolates but a lower *γ* than *L. booriae* (*N* = 126 and 90, *γ =* 0.159 and 0.208, respectively), whereas *L. welshimeri* included more isolates (*N* = 141) but exhibit a smaller *γ* (0.133) [10]. Fisher’s exact tests followed by BH-FDR correction were performed to identify lineage-associated genes. Functional enrichment analysis based on a binomial distribution model described in Liao *et al.* [20] was performed to identify COGs significantly overrepresented among lineage-associated genes.

To further identify genomic differences among lineages, we analysed genome size and GC content [10]; virulence factors, including *Listeria* Pathogenicity Island (LIPI-1, -3, -4) and internalin (*inl*) genes; SSI-1 and -2 [20]; and antibiotic resistance genes (ARGs) and mobile genetic elements (MGEs), such as insertion sequences (IS), transposons, prophages, and plasmids [39]. A full list of virulence factors, SSI, and ARGs queried is provided in the Supplementary Information. While these genomic elements were previously reported, they were not compared among *Lm* lineages. Here, KW tests were used to assess the differences in genome size, GC content, and MGEs among lineages, followed by two-sided MW *U* tests with BH-FDR correction to assess the pairwise differences. Fisher’s exact tests with BH-FDR correction were used to compare the frequency of virulence factors, SSI, and ARGs among lineages.

### Identification of epidemiological links between *Lm* soil and clinical isolates

To identify human clinical isolates closely related to our *Lm* isolates, the NCBI Pathogen Detection Isolates Browser [41] was accessed in 2019 to identify human clinical *Lm* isolates found within the same single-linkage single-nucleotide polymorphism (SNP) cluster as with one or more of the 177 soil *Lm* isolates included in this study. In the NCBI Pathogen Detection framework, a single-linkage SNP cluster groups isolates differing by no more than 50 SNPs and is used for genomic surveillance and phylogenetic context [42]. A total of 186 closely related clinical isolates were identified, all of which met the genome assembly quality criteria outlined in the Supplementary Information (see Supplementary Table 2 for quality metrices). To confirm the genetic relatedness between soil-derived and closely related clinical isolates, a maximum likelihood phylogenetic tree was constructed based on core SNPs identified using kSNP4 [43]. The tree was built using RAxML-8.2.13 [44] with the GTR + G model selected by jModelTest [44], incorporating ascertainment bias correction and 1000 bootstrap repetitions, and was visualized using iTOL v6 webserver [45].

To identify epidemiologic links between these closely related soil and clinical *Lm* isolates, core genome multilocus sequence typing (cgMLST) was profiled, in which allelic types for cgMLST genes were assigned based on the database available on the BIGSdb-*Lm* platform [28]. Isolates differing by up to seven allelic mismatches in cgMLST profiles were considered indicative of potential epidemiological links, based on a previous study that confirmed outbreaks typically involve isolates differing by seven or fewer alleles [28]. To assess genomic similarities between soil isolates and its epidemiologically linked clinical isolates if multiple identified, Wilcoxon rank-sum tests were performed to compare their genome size, GC content, and total counts for virulence factors, SSI-1 and -2, ARGs, and normalized abundance of MGEs.

## Results

### Associations between abiotic environmental factors and *Lm* pangenome

**Figure 1:**
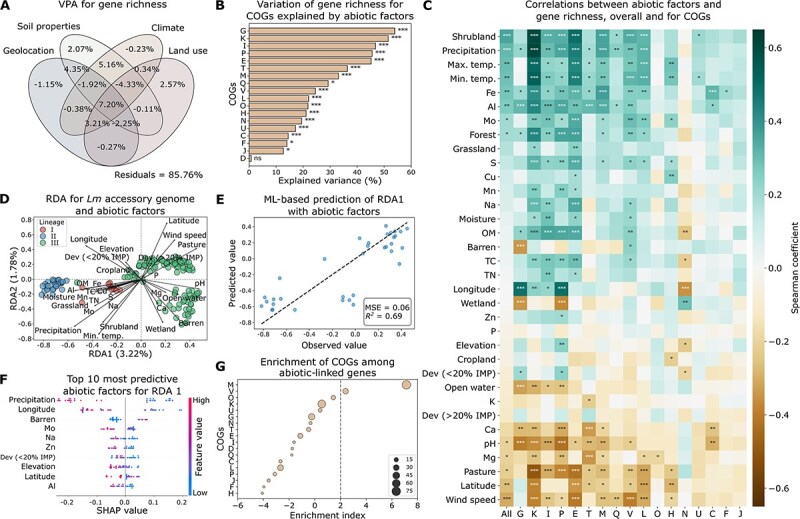
Genomic variation of *Lm* is associated with abiotic factors. (A) Venn diagram of VPA showing the variation of gene richness explained by geolocation, soil properties, climate, and surrounding land use. Residuals represent unexplained variation. A negative explained variation may be caused by collinearity between predictors and overparameterization of the model. (B) Variation of gene richness for COGs explained by abiotic factors in VPA. Statistical significance was assessed using a one-sided permutation test. (C) Spearman’s correlation between abiotic factors and gene richness, overall and for significant COGs identified in VPA. Abiotic factors are ordered by the descending correlation coefficients of overall (“All”) gene richness. Positive and negative correlations are indicated by green and brown, respectively. (D) RDA biplot illustrating the relationships between the *Lm* accessory genome and 31 abiotic variables selected by Lasso regularization. Points represent individual genomes, color-coded by lineages. The length and direction of the arrows indicate the relative importance and correlation of each abiotic variable with the ordination axes. The first (RDA1) and second (RDA2) ordination axes explain 3.22% and 1.78% of the variation of the *Lm* accessory genome explained by selected abiotic factors, respectively. (E) Prediction of RDA1 axis values with abiotic factors using the LightGBM ML model. MSE, mean squared error; *R^2^*, coefficient of determination. The dashed line represents the line of perfect agreement (y = x) where predicted values would exactly match observed values. (F) The top 10 most predictive abiotic factors for RDA1 (SHAP-based; X axis), sorted by descending importance. SHAP values indicate the impact of features on ML model output. (G) Enrichment of COGs among abiotic-linked genes. An enrichment index greater than two (grey dashed line) indicates significant overrepresentation (*P* < .05). Circle size is proportional to the number of genes annotated to each COG. For (B) and (C), significance levels are denoted by “*”, “**”, “***”, and “ns” for adjusted *P* < .05, < .01, < .001, and > .05 (not significant), respectively. For (B), (C), and (G), COGs abbreviations are as follows: C: Energy production and conversion; D: Cell cycle control, cell division, chromosome partitioning; E: Amino acid transport and metabolism; F: Nucleotide transport and metabolism; G: Carbohydrate transport and metabolism; H: Coenzyme transport and metabolism; I: Lipid transport and metabolism; J: Translation, ribosomal structure, and biogenesis; K: Transcription; L: Replication, recombination, and repair; M: Cell wall/membrane/envelope biogenesis; N: Cell motility; O: Posttranslational modification, protein turnover, chaperones; P: Inorganic ion transport and metabolism; Q: Secondary metabolites biosynthesis, transport, and catabolism; T: Signal transduction mechanisms; U: Intracellular trafficking, secretion, and vesicular transport; V: Defense mechanisms.

For the 177 *Lm* soil isolates included in this study, a total of 5873 orthologous genes were identified, with 2324 and 3549 classified as core and accessory genes, respectively (Supplementary Fig. 1). The gene richness per genome ranged from 2627 to 3082 (Supplementary Fig. 1). To identify key abiotic drivers of the pangenome composition of *Lm*, we first performed a series of statistical analyses assessing the relationships between 34 abiotic environmental variables and gene richness. VPA combined with a permutation test (see Methods) revealed that abiotic factors altogether significantly explained 14.24% of the gene richness variation (one-sided permutation *P* = .01; Fig. 1A). Among them, soil property and climatic variables were found to be most influential, accumulatively explaining 10.17% and 9.05% of the variation with other variables, respectively, followed by geolocation (8.79%) and surrounding land use (6.36%; Fig. 1A). Of note, the interplay of soil properties and climate explained 5.16% of the variation (Fig. 1A). Negative explained variations likely reflect collinearity among predictors or model overparameterization [46, 47] and are not biologically meaningful [47]. Stratifying by COGs, abiotic factors were found to significantly explain the variation of gene richness for 17 out of 18 COGs (adjusted one-sided permutation *P* < .05 for all), with G (carbohydrate transport and metabolism) and K (transcription) exhibiting over 50% of the variation explained (Fig. 1B).

To assess the relationships between individual abiotic factors and gene richness, Spearman’s correlation analysis was performed for overall gene richness as well as that for each COG. Precipitation, temperature, soil Al, Fe, and surrounding shrubland coverage were significantly positively correlated with overall gene richness, while wind speed, Mg, pH, and surrounding pasture coverage showed a significant negative correlation (adjusted Spearman’s *P* < .05 for all; Fig. 1C). At the functional level, these abiotic variables were also significantly correlated with gene richness for over half of the 17 significant COGs identified in VPA (adjusted *P* < .05 for all; Fig. 1C; Supplementary Fig. 2), indicating consistent associations across multiple functions. In contrast, some abiotic variables tend to have a function-specific effect on gene richness. For example, K and Zn were only significantly correlated with gene richness for COGs T (signal transduction mechanisms) and P (inorganic ion transport and metabolism), respectively (adjusted *P* < .05 for both; Fig. 1C).

In addition to gene richness, we also assessed the relationships between abiotic variables and the presence/absence of accessory genes using RDA. The 31 out of 34 abiotic variables selected by Lasso regularization were found to explain 3.22% and 1.78% of the variation in the first (RDA1) and second (RDA2) ordination axis, respectively, with precipitation, minimum temperature, wind speed, Al, pH, Mo, latitude, longitude, and surrounding shrubland, pasture, barren, and wetland coverage exerting a strong effect (Fig. 1D). As the relationships between abiotic variables and accessory gene variance may not be captured by the linear constraints of RDA, which could result in the observed low explained variance [48, 49], we further employed ML to model their relationships (see Methods). Results show that with LightGBM and random forests, 69% and 84% of the variation in RDA1 and RDA2 was explained by these selected abiotic variables, respectively (Fig. 1E; Supplementary Fig. 3A). Based on SHAP analysis [40], precipitation, minimum temperature, Al, Mo, latitude, longitude, and surrounding coverage of shrubland and barren, which were found to have a strong effect in the RDA biplot (Fig. 1D), were also among the top 10 most predictive abiotic factors for RDA1 and/or RDA2 (Fig. 1F; Supplementary Fig. 3B). Analyses on gene richness and accessory genomes altogether suggest that the genomic variation of *Lm* is strongly associated with climatic variables (e.g. precipitation and temperature) and soil properties (e.g. Al, pH, and Mo).

To assess the associations between abiotic factors and individual accessory genes, we compared the difference of abiotic factors for *Lm* genomes with and without a given accessory gene. A total of 803 accessory genes were found to be significantly associated with at least one abiotic factor, which we defined as “abiotic-linked genes” (adjusted MW *U P* < .05 for all; Supplementary Table 3). Functional enrichment analysis (see Methods) showed that COGs M (cell envelope biogenesis) and V (defense mechanisms) were significantly enriched among these abiotic-linked genes (Fig. 1G). These results along with gene richness analysis COGs show evidence that abiotic factors play a critical role in structuring the gene content of soil-dwelling *Lm* across multiple functions, particularly for genes involved in cell envelope formation and defense mechanisms.

### Associations between bacterial community composition and *Lm* pangenome

**Figure 2:**
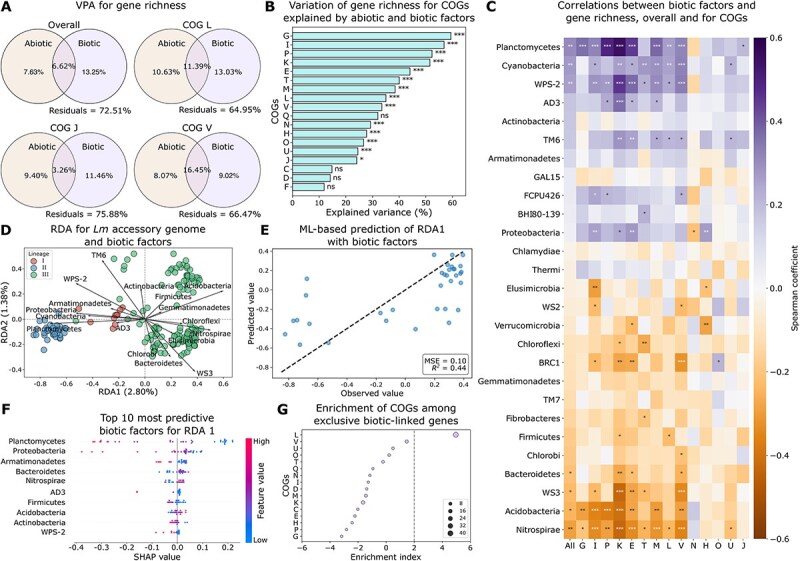
Genomic variation of *Lm* is associated with bacterial community composition. (A) Venn diagram of VPA showing the variation of gene richness explained by abiotic and biotic factors. Biotic factors are represented by bacterial community composition at the phylum level. COGs where biotic factors explain more variation than abiotic factors are highlighted. Residuals represent unexplained variation. (B) Variation of gene richness for COGs explained by abiotic and biotic factors in VPA. Statistical significance was assessed using a one-sided permutation test. (C) Spearman’s correlation between the relative abundance of bacterial phyla and gene richness, overall and for significant COGs identified in VPA. Bacterial phyla are ordered by the descending correlation coefficients of overall (“All”) gene richness. Positive and negative correlations are indicated by purple and orange, respectively. (D) RDA biplot illustrating the relationships between the *Lm* accessory genome and 17 phyla selected by Lasso regularization. Points represent individual genomes, color-coded by lineages. The length and direction of the arrows indicate the relative importance and correlation of each phylum with the ordination axes. RDA1 and RDA2 account for 2.80% and 1.38% of the variation of the *Lm* accessory genome explained by selected phyla, respectively. (E) Prediction of RDA1 axis values with relative abundance of bacterial phyla using random forests. MSE, mean squared error; *R^2^*, coefficient of determination. The dashed line represents the line of perfect agreement (y = x) where predicted values would exactly match observed values. (F) The top 10 most predictive biotic factors for RDA1 (SHAP-based; X axis), sorted by descending importance. SHAP values indicate the impact of features on ML model output. (G) Enrichment of COGs among exclusive biotic-linked genes. An enrichment index greater than two (indicated by the grey dashed line) signifies significant enrichment (*P* < .05). Circle size is proportional to the number of genes annotated to each COG. Abbreviations of COGs are described in the legend of Fig. 1. For (B) and (C), significance levels are denoted by “*”, “**”, “***”, and “ns” for adjusted *P* < .05, < .01, < .001, and > .05 (not significant), respectively. For (C), (D), and (F), WPS-2, TM6, WS3, BRC1, AD3, FCPU426, WS2, and BHI80-139 represent candidate bacterial phyla identified through 16S rRNA sequencing that remain uncultured under laboratory conditions.

*Lm* does not exist in isolation within the soil environment; instead, it coexists within communities alongside other bacteria. Among the soil samples positive for *Lm*, a total of 28 phyla were identified. To test the contribution of bacterial community composition (also referred to as “biotic factors”) to the variation of *Lm* pangenomes, VPA was performed on the gene richness and the relative abundance of bacterial phyla along with abiotic factors. Bacterial phylum composition alone and cumulatively with abiotic factors significantly contributed to 13.25% and 19.87% of the variation of gene richness, respectively (one-sided permutation *P* < .001; Fig. 2A). Stratifying by COGs, the variation of gene richness for 14 COGs was significantly explained by both abiotic factors and bacterial phylum composition (adjusted one-sided permutation *P* < .05 for all; Fig. 2B). Among these COGs, G (carbohydrate transport and metabolism), I (lipid transport and metabolism), P (inorganic ion transport and metabolism), and K (transcription) had over 50% of their variation explained, indicating a significant impact of abiotic and biotic factors on these gene functions (Fig. 2B). Of note, COGs L (replication, recombination, and repair), J (translation, ribosomal structure, and biogenesis), and V (defense mechanisms) showed greater variation of gene richness explained by biotic factors than abiotic factors, suggesting higher importance of bacterial community composition impacting these functions (Fig. 2A).

**Figure 3:**
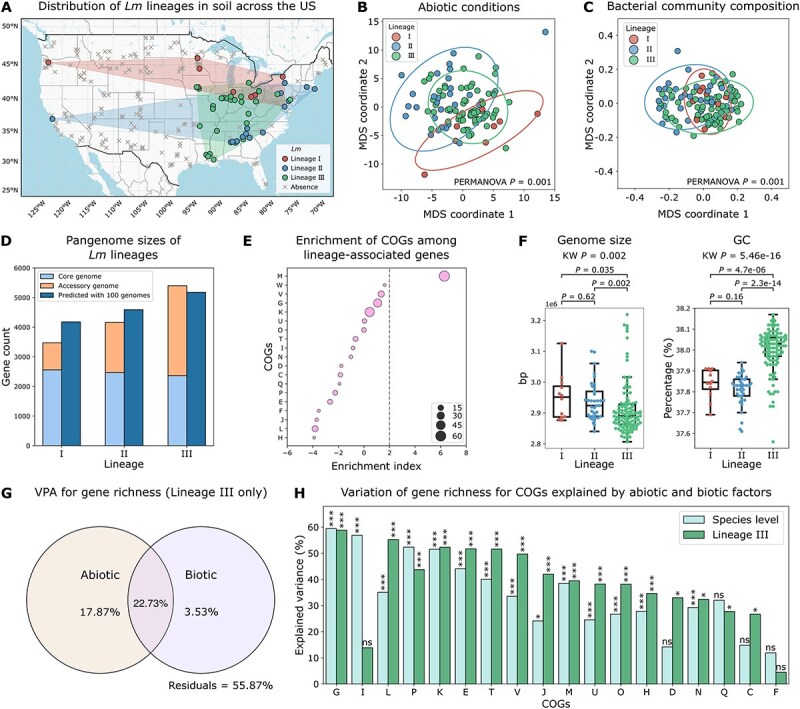
Pangenome variation is substantial among *Lm* lineages. (A) Map showing the distribution of *Lm* lineages in soils across the USA. Points represent *Lm*-positive samples, color-coded by lineages, and gray crosses mark sites where *Lm* was not detected. Polygons indicate the habitat range of each lineage. This map was adapted from **Extended Data Fig. 5** in Liao *et al*. [10]. (B–C) MDS analysis for *Lm* lineages based on (B) abiotic conditions (pseudo-*F* = 6.703) and (C) bacterial community composition using weighted UniFrac distances based on OTUs (pseudo-*F* = 8.829). Points represent *Lm* genomes color-coded by lineages. PERMANOVA *P* = .001 indicates significant differences in abiotic and biotic conditions among lineages. Ellipse size represents two times the standard deviation from the mean. (D) Pangenome sizes of *Lm* lineages, stratified into core and accessory genome sizes, and predicted pangenome sizes based on 100 genomes per lineage. Pangenome size predictions were made using the power law function *cNγ* estimated in **Extended Data Fig. 7** in Liao *et al*. [10]. (E) Enrichment of COGs among lineage-associated genes. An enrichment index greater than two (indicated by the grey dashed line) signifies significant enrichment (*P* < .05). The size of the circles is proportional to the number of genes annotated to each COG. COG abbreviations are described in Supplementary Information. (F) Genome size and GC content compared among *Lm* lineages. Box plots display the interquartile range (IQR) with the median indicated as a line and whiskers extending to 1.5 times the IQR. Adjusted KW *P*-value and adjusted two-sided MW *U P*-values are annotated for each boxplot. (G) Venn diagram of VPA showing the variation of gene richness for lineage III explained by abiotic and biotic factors. Residuals represent unexplained variation. (H) Variation of gene richness for COGs explained by abiotic and biotic factors at the species level (cyan) and within lineage III (green). Significance levels are denoted by “*”, “**”, “***”, and “ns” for adjusted *P* < .05, < .01, < .001, and > .05 (not significant), respectively, and “ns” in one-sided permutation tests for VPA.

To pinpoint specific bacterial phyla associated with gene richness, Spearman’s correlation analysis was performed on the relative abundance of phyla and overall gene richness as well as that for each COG. Results showed that Planctomycetes, Cyanobacteria, and WPS-2 were significantly positively correlated with overall gene richness, while Nitrospirae, Acidobacteria, WS3, and Bacteroidetes showed a significant negative correlation (adjusted Spearman’s *P* < .05 for all; Fig. 2C). WPS-2 and WS3 represent candidate bacterial phyla that remain uncultured under laboratory conditions. At the functional level, Nitrospirae, Planctomycetes, Cyanobacteria, WPS-2, and Acidobacteria were significantly correlated with gene richness for over half of the 14 significant COGs identified in VPA (adjusted *P* < .05 for all; Fig. 2C; Supplementary Fig. 4), indicating consistent associations across multiple functions. In contrast, some phyla tend to have a function-specific effect on gene richness. For example, Fibrobacteres and Chlorobi were only significantly correlated with gene richness for COGs T (signal transduction mechanisms) and V (defense mechanisms), respectively (adjusted *P* < .05 for both; Fig. 2C).

In addition to gene richness, we further examined the relationships between bacterial community composition and the presence/absence of accessory genes. RDA showed that 17 out of 27 phyla selected by Lasso regularization explained 2.80% and 1.38% of the variation in RDA1 and RDA2 of the accessory gene content, respectively, with TM6, Planctomycetes, WS3, WPS-2, Acidobacteria, Proteobacteria, Nitrospirae, Bacteroidetes, Cyanobacteria, and Chlorobi exerting a strong effect (Fig. 2D). ML models further enhanced the prediction, with 44% and 42% of the variation in RDA1 and RDA2 being explained by selected phyla using random forests and gradient boosting, respectively (Fig. 2E; Supplementary Fig. 5A). Based on SHAP analysis [40], TM6, Planctomycetes, WS3, WPS-2, Acidobacteria, Proteobacteria, Nitrospirae, Bacteroidetes, and Cyanobacteria, which were found to have a strong effect in RDA (Fig. 2D), were also among the top 10 most predictive phyla for RDA1 and/or RDA2 (Fig. 2F; Supplementary Fig. 5B). Analyses on gene richness and accessory genes altogether suggest that bacterial community composition, particularly Nitrospirae, Planctomycetes, Acidobacteria, and Cyanobacteria, play an important role in genomic variation of *Lm*.

To assess the associations between biotic factors and individual accessory genes, we compared the relative abundance of phyla for *Lm* genomes with and without a given accessory gene. A total of 807 accessory genes were found to be significantly associated with at least one phylum, referred to as “biotic-linked genes” (adjusted MW *U P* < .05 for all; Supplementary Table 4). Functional enrichment analysis showed that these biotic-linked genes were significantly enriched for cell envelope biogenesis (M) (Supplementary Fig. 6A). Comparing these 807 biotic-linked genes to the 803 abiotic-linked genes, 299 genes (27.0%) and 303 genes (27.4%) were exclusively linked to abiotic factors and biotic factors, respectively (Supplementary Fig. 6B). Functional enrichment analysis showed that COGs M (cell wall/membrane/envelope biogenesis) and V (defense mechanisms) were significantly enriched among genes exclusively linked to abiotic factors (Supplementary Fig. 6C), while L (replication, recombination, and repair) was significantly enriched among genes exclusively linked to biotic factors (Fig. 2G), consistent with the VPA result where biotic factors showed higher importance for gene richness involved in this function (Fig. 2A). These results along with gene richness analysis for COGs suggest that bacterial community composition is important to the gene content of soil-dwelling *Lm* across multiple functions, particularly for genes involved in cell envelope formation jointly with abiotic factors and uniquely in replication, recombination, and repair.

### Pangenome variation among *Lm* lineages

Three *Lm* lineages were detected in soil, with the 177 isolates classified into lineage I (12 isolates), lineage II (39 isolates), and lineage III (126 isolates). Geographically, lineages I and II were more widely distributed, whereas lineage III formed a geographic cluster in the central and eastern areas (Fig. 3A). Among the 34 abiotic variables, 22 were significantly different among the three lineages (adjusted KW *P* < .05 for all; Supplementary Fig. 7). Of note, lineage III appears to be more capable of surviving in nutrient-limited conditions compared to lineages I and II, as its habitats had significantly lower levels of OM, TN, Na, and S (adjusted MW *U P* < .05 for all; Supplementary Fig. 7). In MDS analysis based on the 34 abiotic variables, genomes formed significantly distinct clusters by lineages (PERMANOVA *P* = .001; Fig. 3B), suggesting that *Lm* lineages inhabit significantly different abiotic conditions.

Similarly, we identified significant differences in the bacterial community composition compared among *Lm* lineages. Of the 28 phyla, Nitrospirae, Planctomycetes, Acidobacteria, and Proteobacteria showed significantly different relative abundance among the three lineages (adjusted KW *P* < .05 for all; Supplementary Fig. 8). Compared to other lineages, the relative abundance of Planctomycetes and Proteobacteria was significantly higher in lineage II and lower in lineage III, respectively (adjusted MW *U P* < .05 for all). Acidobacteria and Nitrospirae were significantly more abundant in lineage III than in lineage II (adjusted *P* = 2.0e-06 and 1.3e-06, respectively; Supplementary Fig. 8). MDS analysis showed that genomes formed significantly distinct clusters by lineages based on weighted UniFrac distances of bacterial communities (PERMANOVA *P* = .001; Fig. 3C). Overall, these results show that *Lm* lineages occupy substantially different abiotic and biotic environmental conditions, which may contribute to the genetic differences across lineages.

Indeed, we observed remarkable differences in the pangenome structure among the three lineages. While their core genome sizes were similar (2560, 2468, and 2366 core genes for lineage I, II, and III, respectively), their accessory genome sizes were substantially different (913, 1695, and 3034 accessory genes, respectively; Fig. 3D). To control for sample size, we predicted the pangenome size for each lineage given 100 genomes based on the pangenome curves (see Methods), which was 4179, 4592, and 5178 genes for lineage I, II, and III, respectively (Fig. 3D). Using Fisher’s exact tests, we identified 670 accessory genes significantly associated with lineages (adjusted *P* < .05 for all; Supplementary Table 5), consistent with the RDA result which identified three distinct clusters by lineages based on accessory genes (Fig. 1D). These lineage-associated genes were significantly enriched for M (cell envelope biogenesis) (Fig. 3E). In addition, we compared the genome size, GC content, virulence factors, SSI 1–2, ARGs, and MGEs among lineages. Lineage III had a significantly smaller genome size (adjusted MW *U P* = .035 and .0022, respectively) and higher GC content (adjusted *P* = 4.7e-06 and 2.3e-14, respectively) compared to lineages I and II (Fig. 3F). For virulence genes, *prfA* and *hly* in LIPI-1 [50], *inl* genes [51], LIPI-3 genes [52], and LIPI-4 genes [53] exhibit significant differences in frequency among lineages (adjusted Fisher’s exact *P* < .05 for all; Supplementary Fig. 9A). Of note, SSI-2, which confers resistance to higher pH [26], was significantly overrepresented in lineage III (adjusted Fisher’s exact *P* = 3.0e-11; Supplementary Fig. 9A), consistent with the observation the habitats of lineage III had significantly higher pH compared to lineage II (adjusted MW *U P* = 5.8e-05; Supplementary Fig. 7). In addition, prevalence of IS elements and transposons significantly differed among lineages (KW *P* = 1.2e-13 and 9.8e-08, respectively; Supplementary Fig. 9B). No significant differences were observed for ARGs (adjusted Fisher’s exact test *P* > .05 for all; Supplementary Fig. 9A), prophages (KW *P* = .88; Supplementary Fig. 9B), and plasmids (KW *P* = .07).

To assess the associations between genomic variation and abiotic and biotic factors at the lineage level, we intended to perform VPA on gene richness stratified by lineages. However, none of the variation was explained for lineages I and II likely due to the limited sample sizes (*n* = 12 and 39, respectively). Therefore, we compared the VPA results for lineage III and species-level results. We found that abiotic and biotic factors jointly explained 44.13% of the variation of gene richness for lineage III (one-sided permutation *P* = 0.01; Fig. 3G), which is substantially higher than the 27.49% observed for overall gene richness for *Lm* (Fig. 2A). Furthermore, VPA stratified by COGs identified 16 COGs with gene richness variation significantly explained by abiotic and biotic factors. Notably, G (carbohydrate transport and metabolism), L (replication, recombination, and repair), K (transcription), E (amino acid transport and metabolism), and T (signal transduction mechanisms) each had over 50% of the variation explained by these factors (adjusted one-sided permutation *P* < .05 for all; Fig. 3H). Among the 18 COGs, 13 COGs (72.2%) showed a higher proportion of variation explained in lineage III compared to the species-level results (Fig. 3H). These findings suggest that the observed associations between abiotic and biotic factors and genomic variation in *Lm* are largely attributable to lineage III, which appears to undergo strong adaption to local nutrient-limited conditions.

### Dispersal dynamics of *Lm* lineages

**Figure 4:**
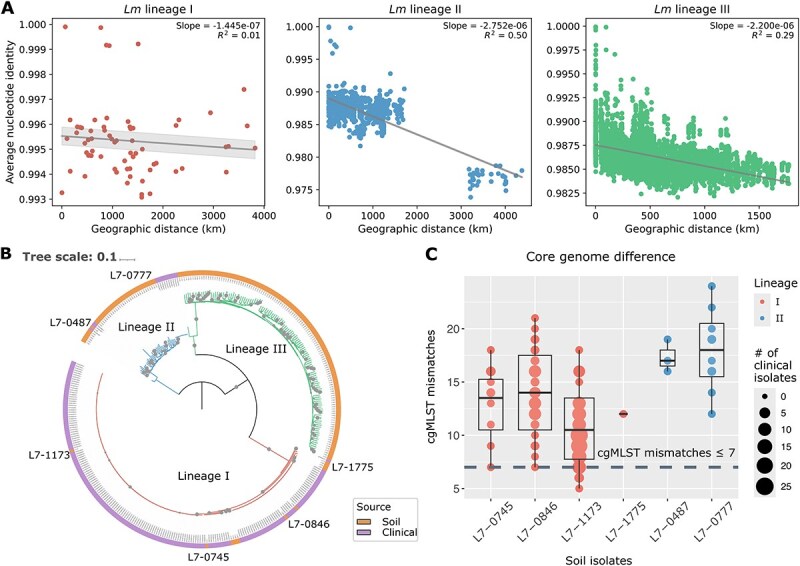
Dispersal patterns of *Lm* lineages in soil and their epidemiological links to clinical isolates. (A) Linear regression for genetic similarities measured by ANI and geographic distances for *Lm* lineage I, II, and III. A steeper negative slope of the fitted regression line with a higher *R^2^* indicates a stronger distance-decay relationship. Shaded areas depict the 95% confidence interval (mean ± 1.96 standard error of mean, SEM) of the linear regression. (B) Maximum likelihood phylogenetic tree based on core SNPs of 177 soil *Lm* isolates (orange) and 186 closely related clinical isolates (purple), with 1000 bootstrap replicates. Bootstrap values >80% are marked by gray circles. The tree is rooted by midpoint, and branches are color-coded by lineages. The six soil isolates with epidemiological links are annotated. (C) cgMLST allelic mismatches between six soil isolates of lineages I (red) and II (blue) and 186 closely related clinical isolates. The horizontal dashed grey line indicates the cutoff (≤ 7 cgMLST mismatches) for documented epidemiological links [28]. The size of the circles is proportional to the number of clinical isolates closely related to a given soil isolate.

Homogenizing dispersal across geographic locations can facilitate evolutionary processes that increase genetic similarity among bacterial populations [15–17]. In contrast, dispersal limitation can enhance differentiation in genetic material across populations that emerge from localized environmental adaptation [15, 16]. To understand the dispersal patterns of *Lm* lineages, we examined the correlations between genetic similarities, measured by ANI, and geographic distances for each lineage. A distance–decay relationship, in which genetic similarities decline with geographic distances, suggests dispersal limitation [54, 55]. Results show that lineage I exhibited no evidence of a distance-decay relationship (*R^2^* = 0.01, slope = 1.45e-07; Fig. 4A), suggesting frequent homogenizing dispersal, in contrast to lineages II and III (*R^2^* = 0.50 and 0.29, slope = −2.75e-06 and − 2.20e-06, respectively; Fig. 4A). To control for potential sample size effects, lineages II and III were downsampled to match the sample size of lineage I, and the patterns remained consistent (*R^2^* = 0.01, 0.62, and 0.31 for lineage I, II, and III, respectively; Supplementary Fig. 10). Notably, the strong distance-decay relationship in lineage II appears to be driven by a single geographically distant isolate observed in the western US. After excluding this isolate, the *R^2^* value for the distance–decay relationship decreased from 0.50 to 0.05 (Supplementary Fig. 11). These results suggest that *Lm* lineage I experience frequent homogenizing dispersal at a nationwide scale, which could reduce genetic differentiation between populations through the transfer of genetic material; the dispersal of lineage II is scale-dependent, where the long-distance dispersal is limited, while regionally (e.g. in the eastern USA), the dispersal is not spatially constrained; and lineage III undergo strong dispersal limitation. These distinct dispersal patterns across *Lm* lineages may partially explain the highest pangenome variation observed in lineage III, followed by lineage I, with lineage II showing the lowest variation. The important role of dispersal in pangenome variation is also supported by the strong correlations observed between geolocation and gene richness (Fig. 1A) and accessory genome composition (Fig. 1D and F).

Given the evidence of frequent homogenizing dispersal observed in *Lm* lineage I, we hypothesized that this lineage may have a higher risk of transmission from soils to humans. To test this, we compared the genomes of our soil isolates with clinical isolates from human infections. A total of 186 clinical isolates were found to be closely related to six soil isolates (<50 SNPs) (Supplementary Figs 12 and 13; Supplementary Table 6). Among these clinical isolates, 173 and 13 were closely related to four soil isolates of lineage I and two soil isolates of lineage II, respectively (Fig. 4B; Supplementary Figs 12 and 13; Supplementary Table 6). Subsequent analysis using cgMLST identified 17 clinical isolates with seven or fewer allelic mismatches with three soil isolates of lineage I, suggesting epidemiological links (Fig. 4C) [28]. Genomic comparisons, including genome size, GC content, virulence factors, ARGs, and MGEs, between these three soil isolates and their corresponding linked clinical isolates further supported the epidemiological links, as no significant differences were identified (Wilcoxon rank-sum *P* > .05 for all; Supplementary Fig. 14). None of the clinical and soil isolates compared contain any SSI or plasmids. These findings indicate a high risk of transmission from soils to humans in *Lm* lineage I.

## Discussion

With in-depth, large-scale pangenome analysis, this study provides evidence that genome flexibility in soil-dwelling *Lm* is jointly influenced by environmental selection and dispersal dynamics. Key abiotic factors that may shape gene gain and loss in *Lm* were climate (e.g. precipitation, temperature, and wind speed) and soil properties (e.g. Al, pH, and Mo). While these abiotic factors tend to affect gene richness across multiple functions (e.g. carbohydrate, lipid, and inorganic ion transport and metabolism and transcription), the pronounced effects were detected among genes involved in cell envelope synthesis and defense mechanisms. The influence of precipitation may be attributed to fluctuations that drive alternating wet–dry cycles in soil, exposing bacteria to both osmotic and desiccation stress. To maintain cellular homeostasis under such conditions, *Lm* may gain or lose genes to modify its membrane composition and structure, as observed in *Bacillus subtilis, Arthrobacter chlorophenolicus,* and *Mycobacterium pallens* [56, 57]. Enrichment in defense-related genes may further facilitate survival under these stresses by encoding stress response proteins that protect against desiccation and oxidative stress, as observed in *Escherichia coli* [58] and *Leptospirillum ferriphilum* [59]. Similarly, rising temperature increases membrane fluidity and permeability [60], prompting homeoviscous adaptation through adjustment in fatty acid composition that counteract temperature-induced changes in lipid packing [56, 60, 61]. In addition, wind can facilitate microbial dispersal [62–64], potentially enabling gene flow among spatially separated populations and thereby contributing to genome diversity. For key soil properties, Al may influence gene gain and loss in combination with soil pH, as under acidic conditions (pH < 5.5), it becomes solubilized into its bioavailable form, which has been shown to impose selective pressure on microbial populations [65–67]. Moreover, Mo, previously identified as an important environmental factor shaping *Listeria* distribution and posing selective pressure on genes encoding the molybdopterin cofactor [10, 68], was also found to be a critical stressor for gene gain and loss in *Lm*. Of note, many of these climatic and edaphic variables are intercorrelated (e.g. temperature, wind speed, precipitation, moisture, and Al) as revealed by our previous analysis [10]. These key abiotic factors may establish broad environmental gradients that jointly influence the genomic variation of *Lm*.

In addition to abiotic factors, we also found evidence that bacterial community composition, particular Nitrospirae, Planctomycetes, Acidobacteria, and Cyanobacteria, plays a role in the genomic variation of *Lm*. Nitrospirae, commonly found in oligotrophic environments [69, 70], are key players in nitrification [71]. Nitrite have been found to inhibit the growth of *Lm* [72–74], which may select genetic machinery that involves retaining, modulating, or losing genes in response to the adverse effects. Planctomycetes has been observed co-existing with *Lm* in some environments, such as compost [75] and spinach leaves [76], while a negative interaction between *Lm* and Acidobacteria has been reported [20, 77, 78]. While the understanding of the interactions between Cyanobacteria and *Lm* is limited, it is known that Cyanobacteria could increase the production cyanotoxins (e.g. microcystins and anatoxin) to gain a competitive advantage under nitrogen-/phosphate-imbalance conditions or in the presence of co-occurring microbial species [79–81]. Like abiotic factors, bacterial community composition was associated with genes across multiple functions, with pronounced effects on genes involved in cell envelope synthesis, in addition to replication, recombination, and repair. Bacterial membranes often house signal transduction systems and histidine kinases crucial for communication, such as the Agr quorum-sensing system [82–84]. Biotic interactions within the community may expose *Lm* to stress that influences the genetic exchange and modulation of genes involved in sensing environmental cues and coordinate their behaviors within the community.

Within-species variation can arise from mutations and gene flow driven by environmental pressures [85]. In *Lm*, substantial pangenome variation was observed among lineages, likely reflecting differences in local environmental adaptation and dispersal dynamics. Lineage III, was found to have a highly open pangenome but streamlined individual genome size with high GC content, which we attributed to strong adaptation to nutrient-limited conditions (e.g. TC and TN). This is consistent with previous finding that smaller genomes with higher GC content may lower the reproductive cost in carbon-limited soils in bacteria [86]. Streamlined genomes enable efficient energy use while preserving the flexibility needed to thrive in challenging ecosystems, balancing adaptability with metabolic efficiency [87, 88]. In response to varying selective pressures encountered in soils, lineage III populations may lose different sets of genes, which along with strong dispersal limitation, has led to its high genomic diversity at the lineage level. In contrast, lineage I, which is commonly associated with foodborne outbreaks [20, 89–92], had a more conserved pangenome, likely maintained by frequent homogenizing dispersal. Similar patterns have been observed in other foodborne pathogens, such as *Vibrio parahaemolyticus* and *Vibrio fluvialis*, where effective gene flow facilitated by frequent dispersal contribute to limited divergence and greater population coherence [93, 94]. Consistent with this strong dispersal potential, lineage I, alongside with II, dominates human clinical and food-associated environments worldwide, including in Brazil, China, Europe, and Norway, with established epidemiological links across North America and Europe indicating substantial long-distance connectivity [28, 53, 89–92]. As previous studies had primarily focused on anthropogenic settings rather than natural environments, our analysis extends this perspective by suggesting that the strong dispersal potential of lineage I may facilitate movement from soils to nearby agricultural fields and/or food processing facilities [20, 28], eventually infecting humans. This transmission route is supported by the epidemiological links between soil-derived and clinical isolates observed in this study as well as links between soil and food-processing isolates reported previously [20], underscoring the need for lineage-specific controlling.

In summary, we provide critical insights into the ecological mechanisms that shape the bacterial genome flexibility and suggest that environmental disturbance, especially shifts in climate patterns, soil properties, and bacterial community composition, can profoundly affect the gene gain and loss process in a bacterial pathogen within its environmental reservoir. To build upon these findings, future studies should aim to experimentally validate these associations through controlled environment experiments, co-culturing with candidate taxa, and deep metagenomic profiling of microbial communities. Particularly, genes involved in cell surface, defense mechanism, and replication, recombination, and repair appear particularly responsive to environmental changes, serving as promising targets for future studies aimed at elucidating the functional roles of flexible genomic regions through gene-cluster and synteny analyses. While our study covers a broad spatial scale, it compromises regional resolution; increasing field sampling density would improve the geographic representativeness of sample sizes across lineages. We also highlight the important role of dispersal across geographic locations, along with environmental selection, in shaping within-species genomic variation. Furthermore, our findings offer critical public health implications, emphasizing the need for targeted genomic monitoring of *Lm* lineages in natural environments adjacent to agricultural areas to enhance source tracking of foodborne outbreaks and better manage transmission risks.

## Study funding

This work was funded by the 4-VA (J.L.) and the Virginia Tech Center for Emerging, Zoonotic, and Arthropod-borne Pathogens (CeZAP) Interdisciplinary Graduate Education Program in Infectious Disease (ID IGEP) fellowship (Y.-X.G.).

## Code and data availability

The whole genome sequencing data for 177 *Lm* soil isolates used in this study have been submitted to the NCBI BioProject database under accession numbers PRJNA514286 and PRJNA561882, along with associated environmental data, which were previously published in Liao *et al*. [10]. The 16S rRNA gene amplicon sequencing data used in this study have been submitted to the NCBI BioProject database under accession number PRJNA749132 and were published in Liao *et al*. [20] NCBI GenBank accession numbers of the genomic assemblies for clinical Lm isolates identified via the NCBI Pathogen Detection Isolates Browser are provided in Supplementary Table 2. All data needed to evaluate the conclusions in the paper are present in the paper and/or the Supplementary Materials. Code to replicate all analyses is publicly available at https://github.com/leaph-lab/Lm_pangenome_MS.

## Supplementary Material

Supplementary_material_ycag093
