# Supplementary material for: Multi-scale ecological drivers shape the genome flexibility of soil-dwelling Listeria monocytogenes
Source: ISME Commun. 2026 Apr 10;6(1):ycag093. doi: 10.1093/ismeco/ycag093 (PMC13155117; doi:10.1093/ismeco/ycag093)
Supplement: Supplementary_material_ycag093 [file supplementary_material_ycag093.zip › Supplementary_Info_isme_commun.pdf]

Supplementary Information

Multi-scale ecological drivers shape the genome flexibility of soil-dwelling *Listeria monocytogenes*

Ying-Xian Goh, FNU Hardeep, Hailong Zhang, Jingqiu Liao

Supplementary Methods

Quality control matrices for genome assemblies

Genome assemblies of isolates used in this study were of high quality, with < 300 contigs, an N50 > 50,000, average coverage > 30X, consistent presence of the *sigB* allelic type in both whole genome and PCR-based assays, no detected contamination using Kraken2 2.0.8 [1], and high genome completeness of 99.99% ± 0.01% (mean ± standard deviation, SD) assessed by CheckM2 [2].

Permutation tests for variation partitioning analysis (VPA)

Gene richness was randomly shuffled across genomes 100 times to generate a null distribution for VPA and the resulting explained variation (i.e., expected explained variation) was compared against the observed explained variation using a one-sided test, with significance set at $P < 0.05$.

Redundancy analysis (RDA)

To select between RDA and canonical correspondence analysis (CCA) for assessing relationships between abiotic/biotic variables and accessory genomes, we performed detrended correspondence analysis (DCA). The first DCA axis length was 0.025, shorter than three standard deviations, indicating that the data is relatively homogeneous and that linear methods, like RDA, are more appropriate for our dataset. Prior to conducting RDA, abiotic factors were standardized to account for the different units of measurement. L1 regularization (Lasso) was applied for feature selection to reduce multicollinearity and enhance model interpretability. DCA and RDA were performed using the vegan package 2.6-4 in R and the skbio library in Python 3.6.8, respectively.

Reference genes retrieved from the BIGSdb-*Lm* platform

Reference virulence factors include LIPI-1 (*prfA, plcA, hly, mpl, actA, plcB*), LIPI-3 (*llsAGHXBYDP*), LIPI-4 (*LM9005581_70009 - LM9005581_70014*), and *inl* genes (*inlAB, C, E, F, G, H, J, K, I, P*). Reference SSI-1 include *lmo0444 - lmo0448* and SSI-2 include *lin0464 - lin0465*. Reference ARGs includes *aacA4, aadC, aadE, aphA, cat_CHL, dfrD, dfrK, ermB, ermG, fexA, fosX, lin, mprF, lnuA, lnuG, mefA, mphB, msrD, norB, penA, qnrB, str, sul, tetM,* and *tetS*.

Supplementary Figures

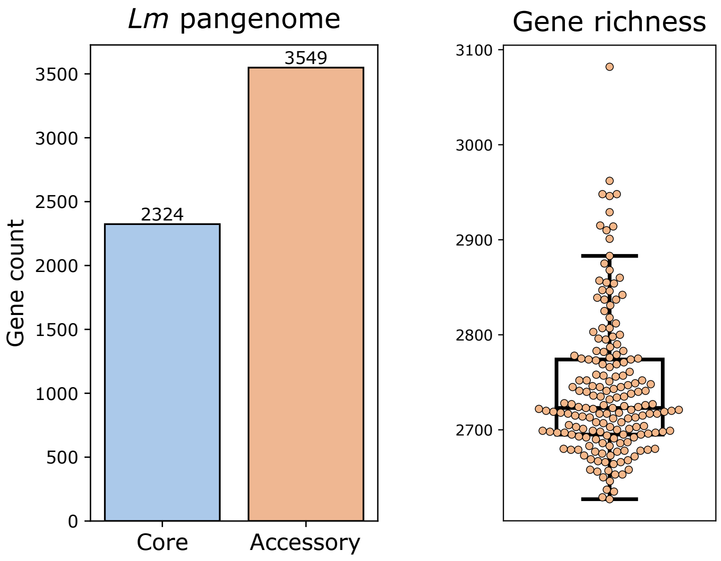

Supplementary Figure 1. Pangenome size and gene richness of *Lm*. Pangenome size of *Lm*, stratified into core and accessory genome sizes, and overall gene richness of *Lm*. Box plot displays the interquartile range (IQR) with the median indicated as a line and whiskers extending to 1.5 times the IQR.

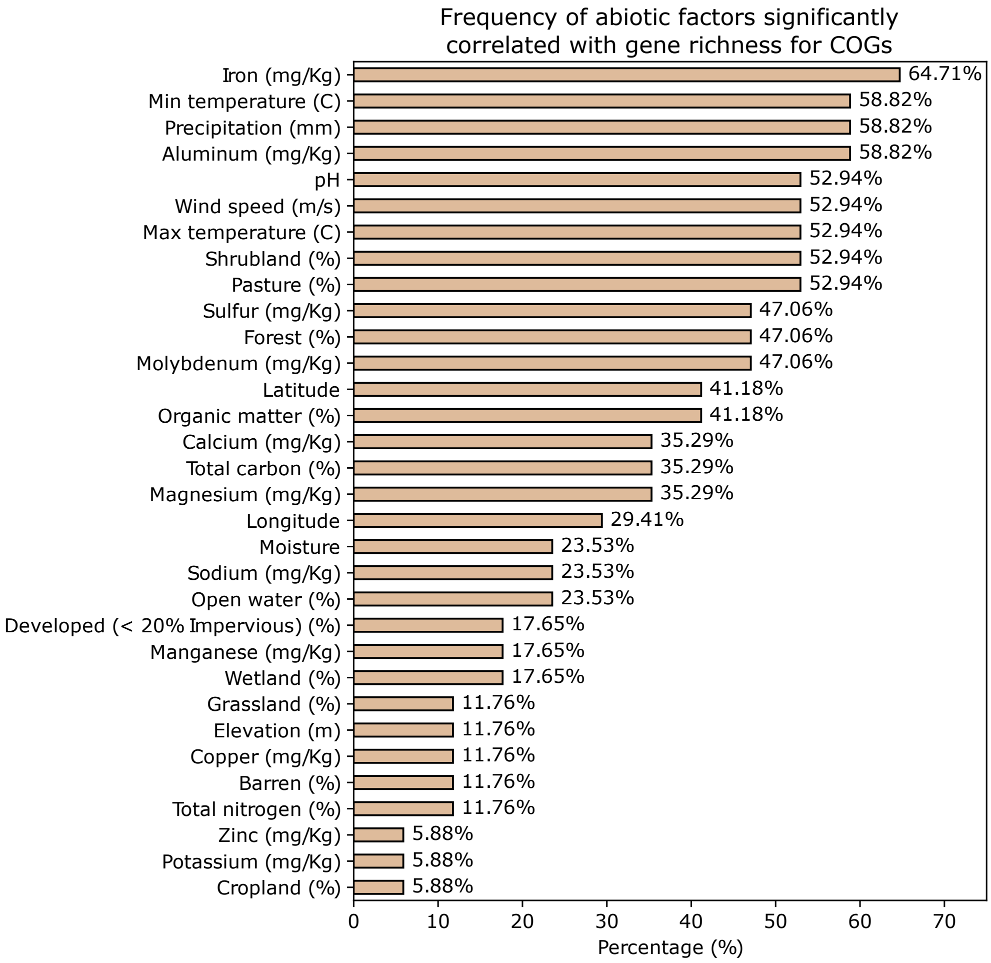

Supplementary Figure 2. Frequency of abiotic environmental factors significantly correlated with gene richness for Clusters of Orthologous Groups (COGs), sorted in descending order. Percentage is calculated as the proportion of COGs for which gene richness was significantly correlated with a given abiotic factor among 17 COGs (adjusted Spearman's $P < 0.05$; correlation analysis results are shown in **Fig. 1C**).

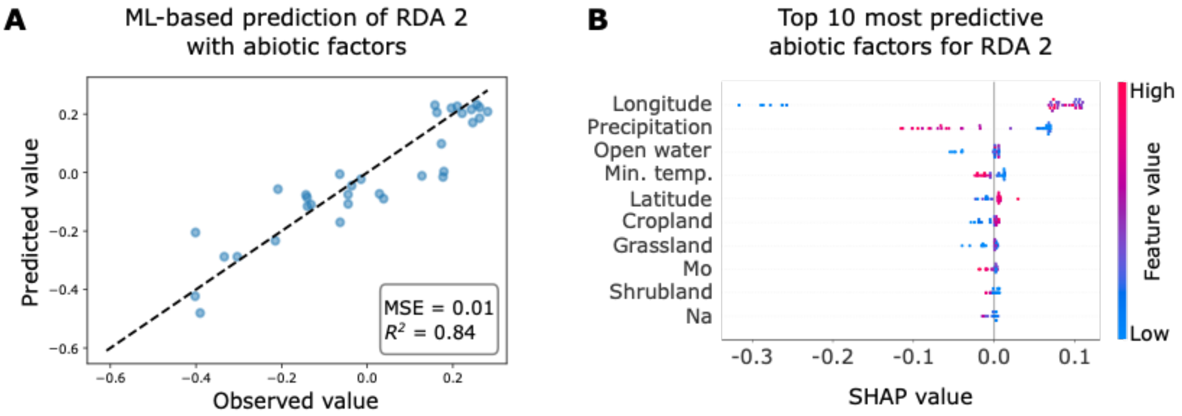

Supplementary Figure 3. **Machine learning (ML) prediction of RDA2 with abiotic factors. (A)** Prediction of RDA2 axis values with abiotic factors using the random forest ML model. MSE, mean squared error; R^2, coefficient of determination. The dashed line represents the line of perfect agreement (y = x) where predicted values would exactly match observed values. **(B)** The top ten most predictive abiotic factors for RDA2 (Shapley Additive exPlanations, SHAP-based; X axis), sorted by descending importance. SHAP values indicate the impact of features on ML model output.

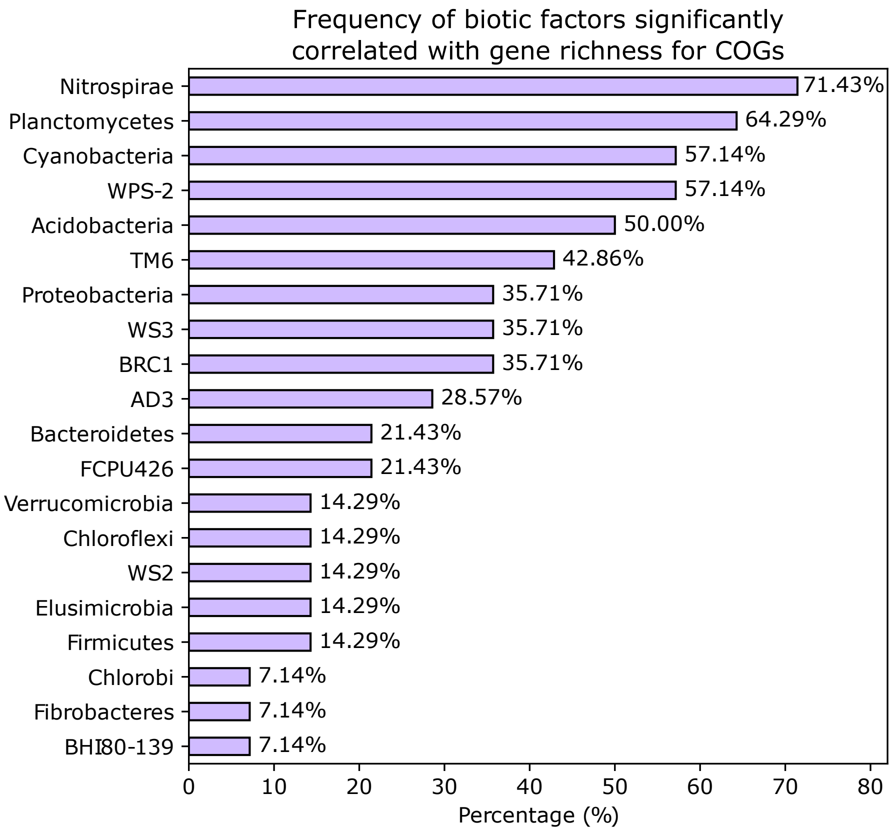

Supplementary Figure 4. Frequency of biotic factors significantly correlated with gene richness for COGs, sorted in descending order. Percentage is calculated as the proportion of COGs for which gene richness was significantly correlated with a given bacterial phylum among 14 COGs (adjusted Spearman's $P < 0.05$; correlation analysis results are shown in **Fig. 2C**). WPS-2, TM6, WS3, BRC1, AD3, FCPU426, WS2, and BHI80-139 represent candidate bacterial phyla identified through 16S rRNA sequencing that remain uncultured under laboratory conditions.

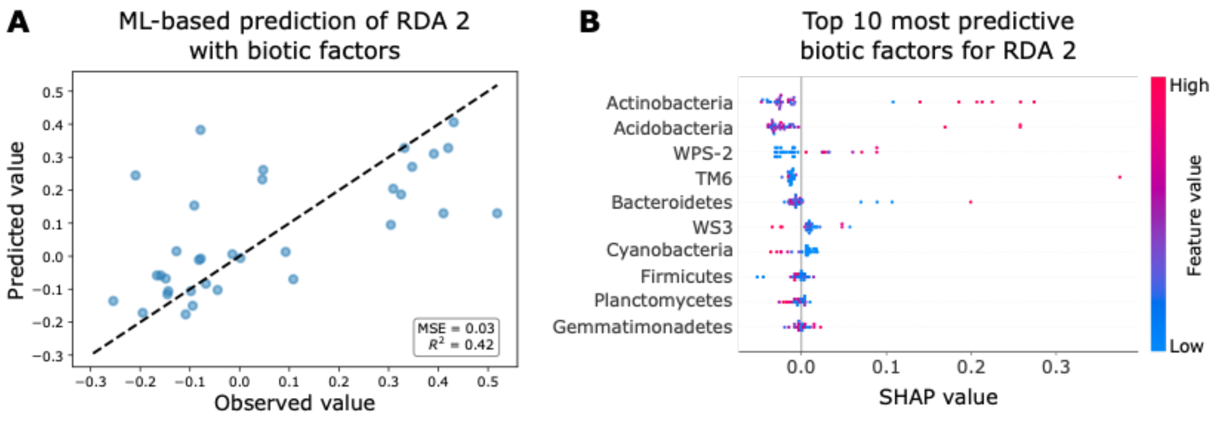

Supplementary Figure 5. ML-based prediction of RDA2 with biotic factors. (A) Prediction of RDA2 axis values with relative abundance of bacterial phyla using a gradient boosting model. MSE, mean squared error; R^2, coefficient of determination. The dashed line represents the line of perfect agreement (y = x) where predicted values would exactly match observed values. **(B)** The top ten most predictive biotic factors for RDA2 axis values (SHAP-based; X axis), sorted by descending importance. SHAP values indicate the impact of features on ML model output. WPS-2, TM6, and WS3 represent candidate bacterial phyla identified through 16S rRNA sequencing that remain uncultured under laboratory conditions.

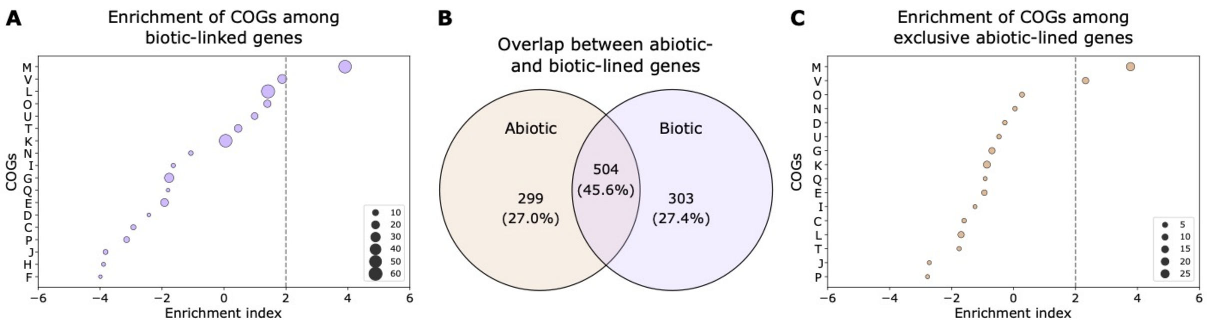

Supplementary Figure 6. Functions of abiotic- and biotic-linked genes. (A) Enrichment of COGs among biotic-linked genes. **(B)** Venn diagram showing the overlap between abiotic- and biotic-linked genes. **(C)** Enrichment of COGs among exclusive abiotic-linked genes. For **(A)** and **(C)**, an enrichment index greater than two (indicated by the grey dashed line) signifies significant enrichment ($P < 0.05$). The size of the circles is proportional to the number of genes annotated to each COG. Abbreviations of COGs are described in Methods.

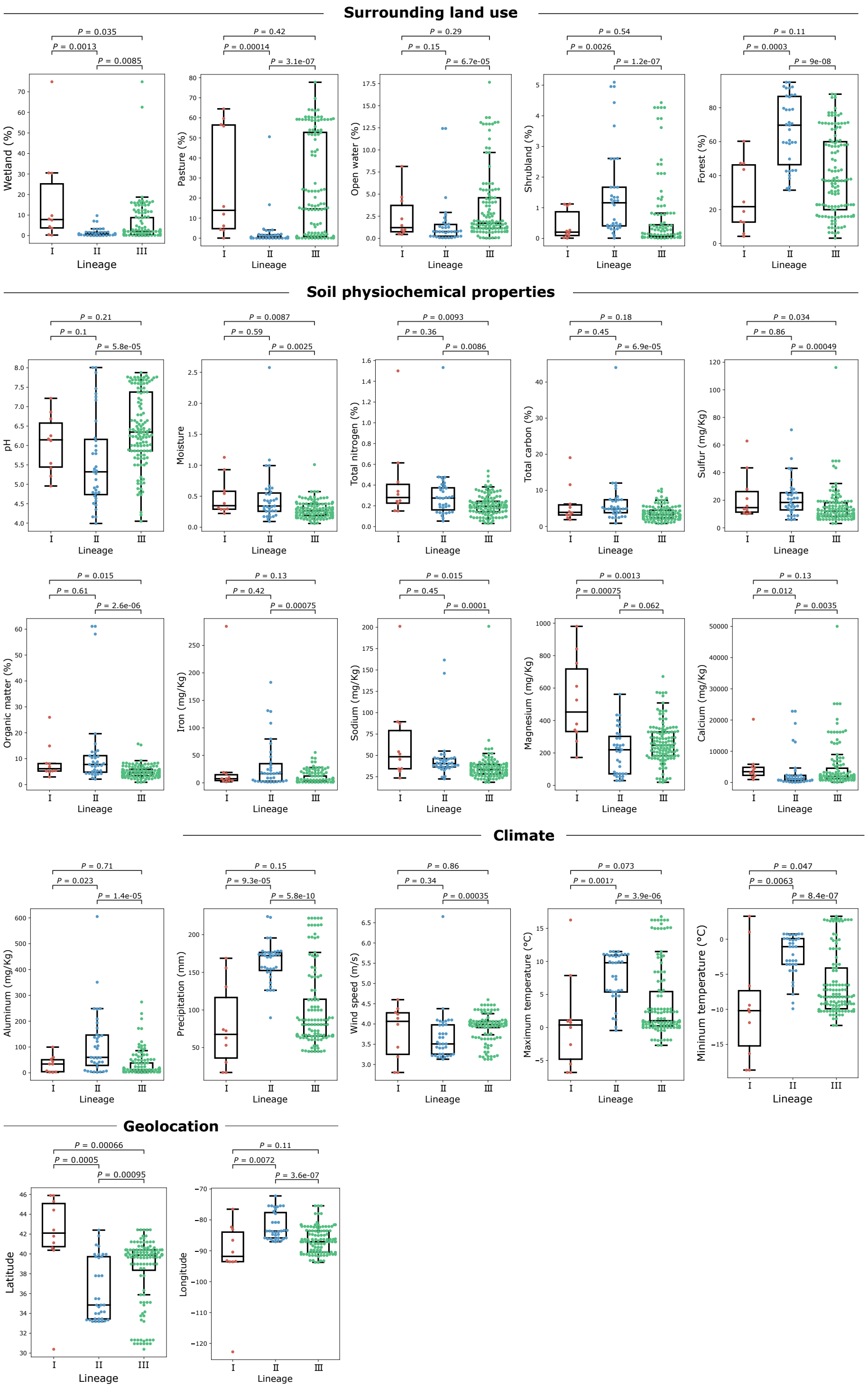

Supplementary Figure 7. Abiotic factors compared among *Lm* lineages. Abiotic factors significantly differing among *Lm* lineages (adjusted Kruskal Wallis [KW] $P < 0.05$) are shown. Boxplots display the IQR with the median indicated as a line and whiskers extending to 1.5 times the IQR. Adjusted two-sided Mann-Whitney (MW) U P values are annotated in each boxplot for pairwise comparison.

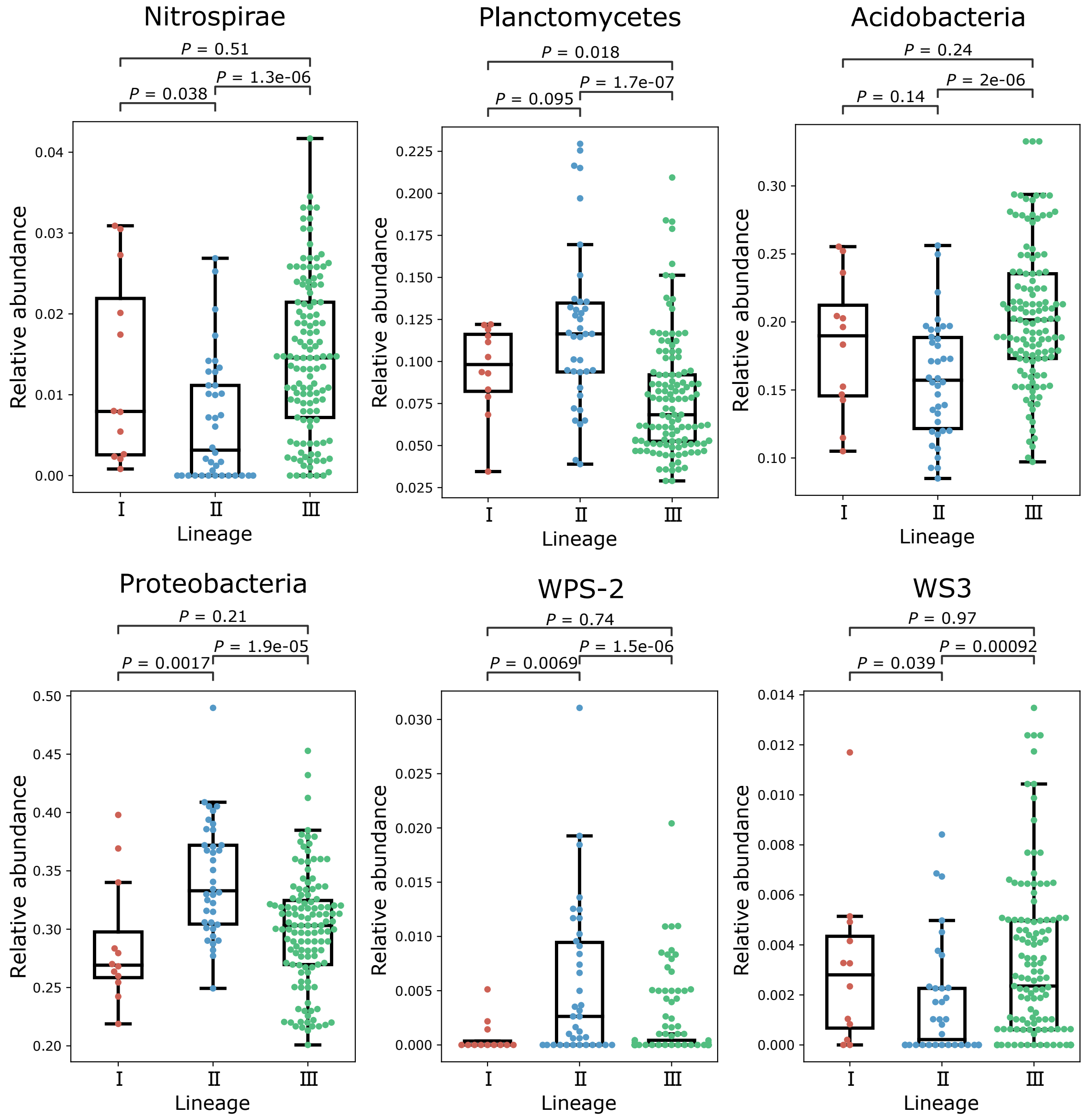

Supplementary Figure 8. Relative abundance of bacterial phyla compared among *Lm* lineages. Phyla significantly differing among *Lm* lineages (adjusted KW $P < 0.05$) are shown. WPS-2 and WS3 represent candidate bacterial phyla identified through 16S rRNA sequencing that remain uncultured under laboratory conditions. Boxplots display the IQR with the median indicated as a line and whiskers extending to 1.5 times the IQR. Adjusted two-sided MW *U P* values are annotated in each boxplot for pairwise comparison.

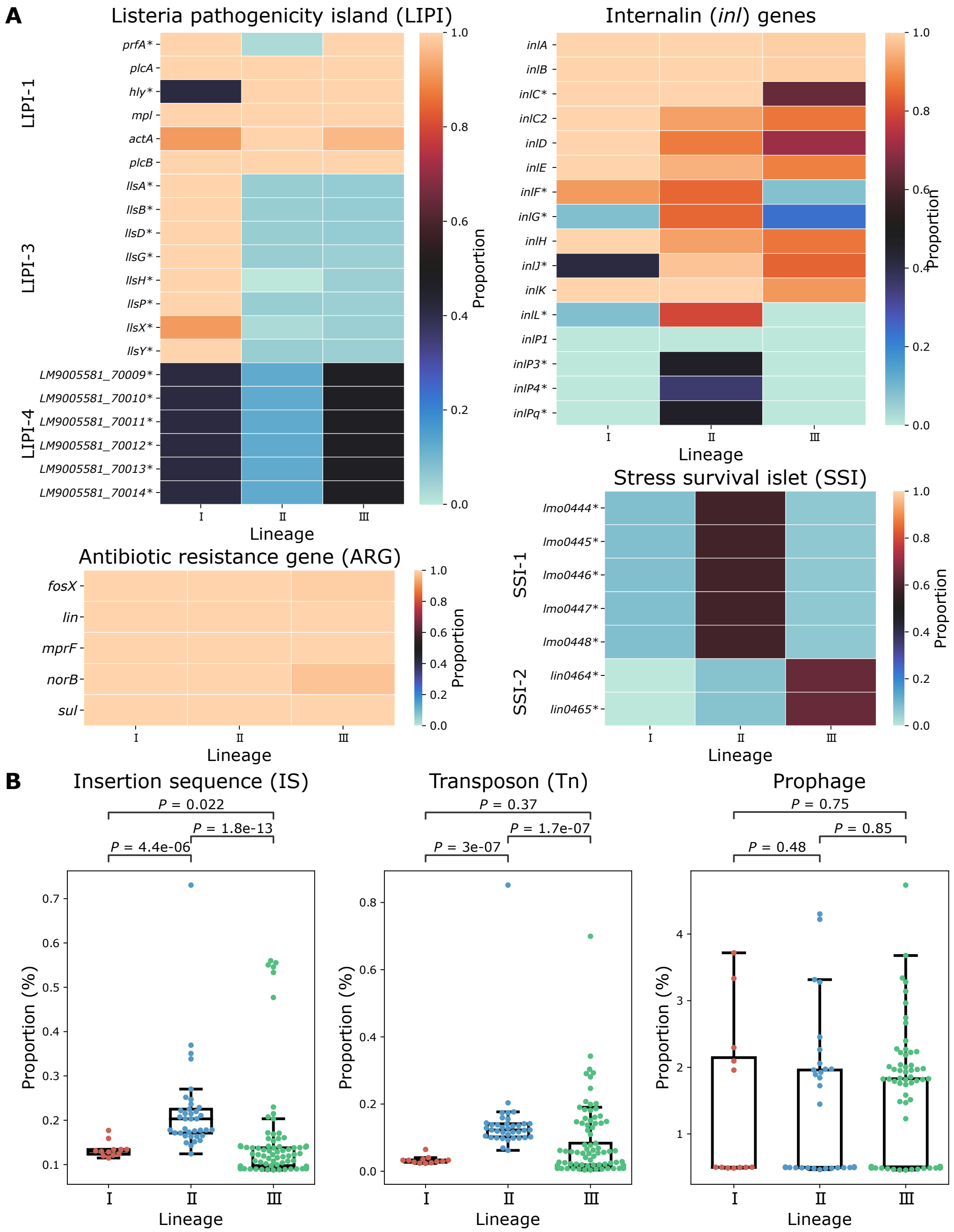

Supplementary Figure 9. Genetic elements compared among *Lm* lineages. (A) Prevalence of *Listeria* pathogenicity island (LIPI)-1, -3, and -4 genes, internalin (*inl*) genes, stress survival islet (SSI) 1-2, and antibiotic resistance genes (ARGs) compared among *Lm* lineages. Significance is denoted by '*' for adjusted *P* < 0.05 in a Fisher's exact test. **(B)** Proportion of insertion sequences (IS), transposons, and prophages compared among *Lm* lineages. Boxplots display the IQR with the median indicated as a line and whiskers extending to 1.5 times the IQR. Adjusted two-sided MW *U P* values are annotated in each boxplot for pairwise comparison.

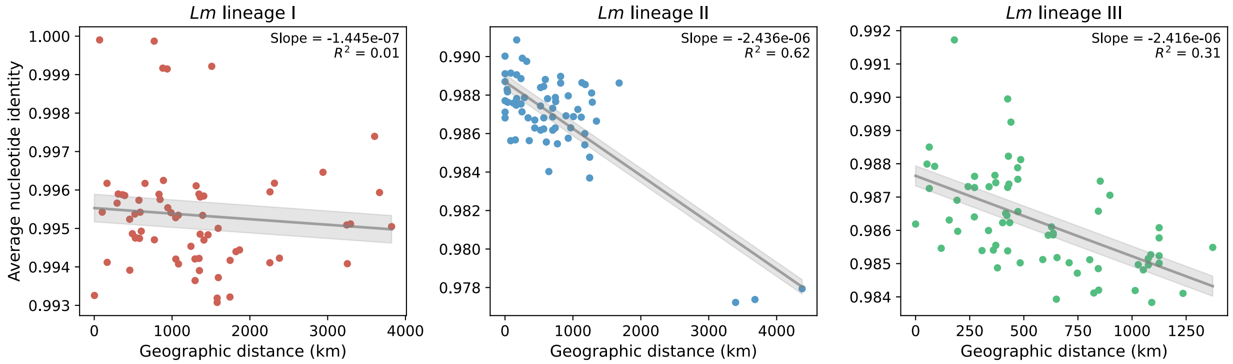

Supplementary Figure 10. Dispersal patterns of *Lm* lineages based on subsampling. Data points for lineages II and III were randomly subsampled to match the sample size of lineage I. Linear regressions of genetic similarity, measured by average nucleotide identity, against geographic distance for lineages I, II, and III. A steeper negative slope of the fitted regression line with a higher R^2 indicates a stronger distance-decay relationship. Shaded areas represent the 95% confidence interval (mean ± 1.96 × standard error of the mean, SEM) of the regression.

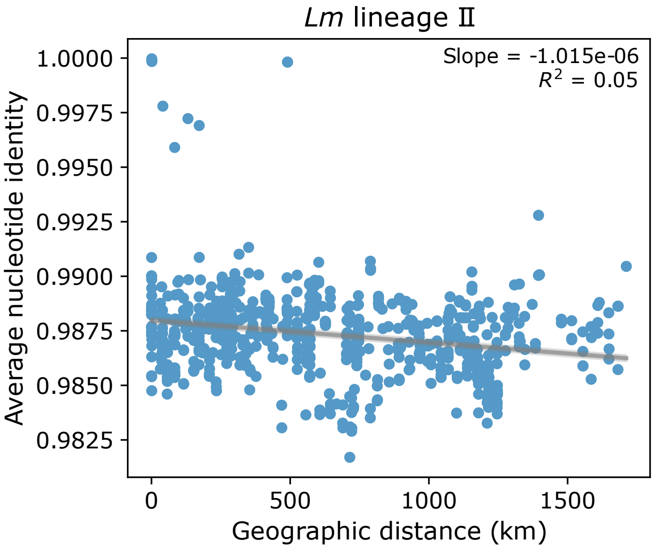

Supplementary Figure 11. Dispersal patterns of *Lm* lineage II after excluding the distant cluster. Linear regressions of genetic similarity, measured by average nucleotide identity, against geographic distance. A steeper negative slope of the fitted regression line with a higher R^2 indicates a stronger distance-decay relationship. Shaded areas represent the 95% confidence interval (mean ± 1.96 × standard error of the mean, SEM) of the regression.

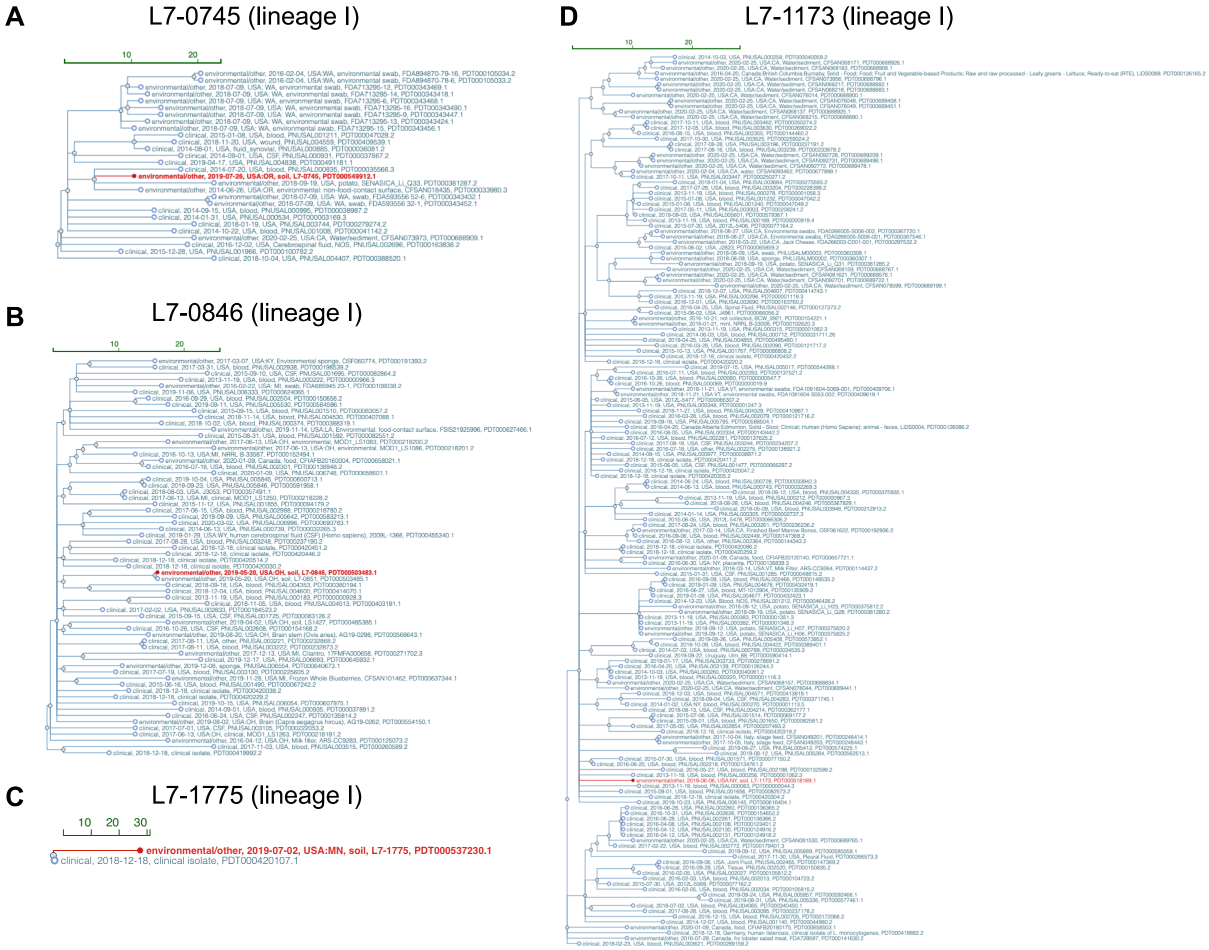

Supplementary Figure 12. Single-linkage single nucleotide polymorphism (SNP) clusters of closely related soil and clinical *Lm* lineage I isolates. (A-D) Clinical *Lm* isolates that formed SNP clusters (< 50 SNPs) with *Lm* lineage I soil isolates **(A)** L7-0745, **(B)** L7-0846, **(C)** L7-1775, and **(D)** L7-1173 in this study. Soil isolates are highlighted in red. Trees were downloaded from NCBI Pathogen Detection Isolates Browser.

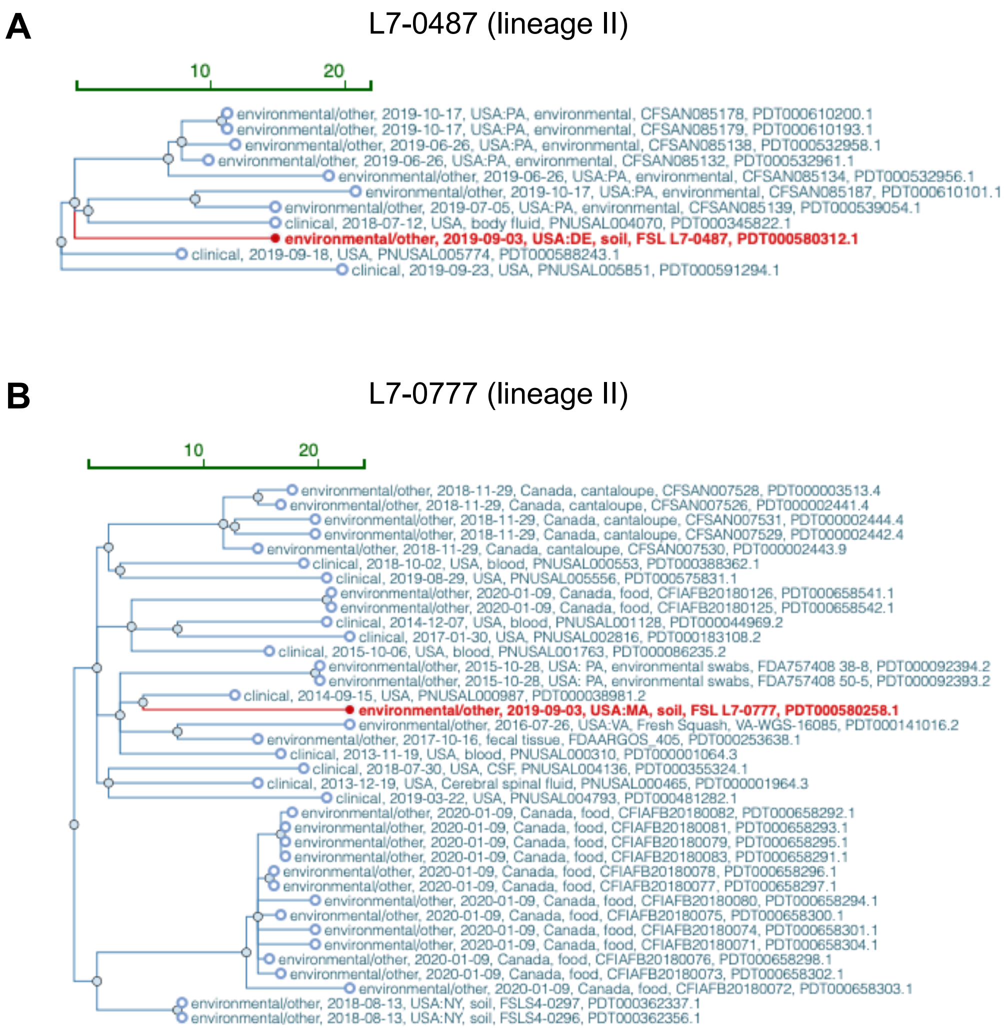

Supplementary Figure 13. Single-linkage SNP clusters of closely related soil and clinical *Lm* lineage II isolates. (A-B) Clinical *Lm* isolates that formed SNP clusters (< 50 SNPs) with *Lm* lineage II soil isolates **(A)** L7-0487 and **(B)** L7-0777 in this study. Soil isolates are highlighted in red. Trees were downloaded from NCBI Pathogen Detection Isolates Browser.

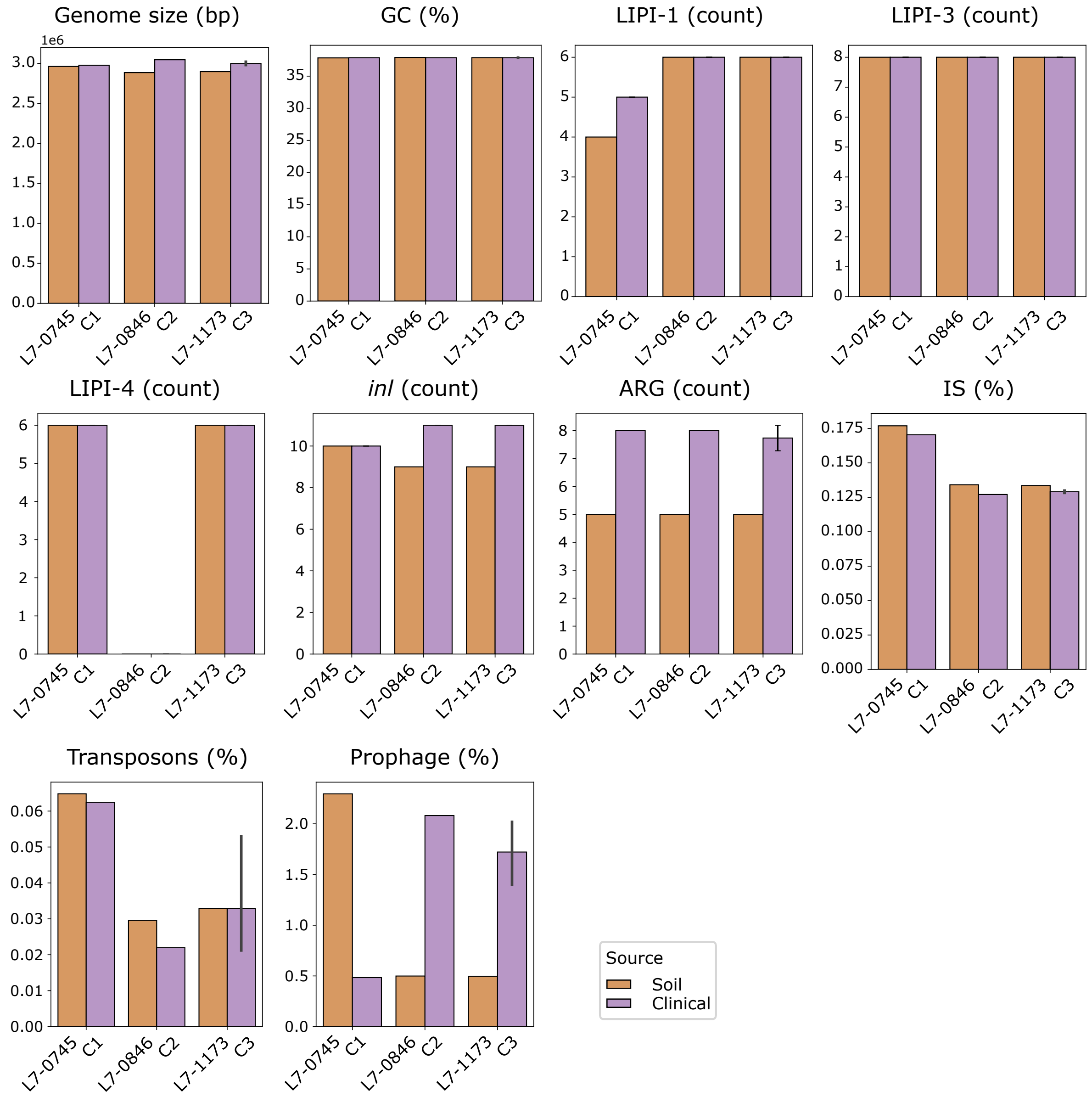

Supplementary Figure 14. Genomic features compared between epidemiologically linked soil and clinical *Lm* lineage I isolates. Genomic features, including genome size, GC content, gene counts for LIPI-1, LIPI-3, LIPI-4, *inl*, and ARGs, as well as the proportions of IS, transposons, and prophages, are compared between soil isolates (L7-0745, L7-0846, and L7-1173; orange) and their epidemiologically linked clinical isolates (C1–C3, respectively; purple). Error bars represent the standard deviation.

Supplementary Tables for this manuscript include the following:

Supplementary Table 1. Hyperparameters for machine learning models.

Supplementary Table 2. List of clinical *Lm* isolates closely related to soil isolates included in this study, identified via NCBI Pathogen Detection Isolates Browser.

Supplementary Table 3. List of abiotic-linked genes, arranged in ascending order of *P*-values.

Supplementary Table 4. List of biotic-linked genes, arranged in ascending order of *P*-values.

Supplementary Table 5. List of lineage-associated accessory genes and their respective *P*-values identified through Fisher's exact test.

Supplementary Table 6. cgMLST differences between soil and clinical *Lm* isolates.

Reference

1. Wood DE, Salzberg SL. Kraken: ultrafast metagenomic sequence classification using exact alignments. *Genome Biol* 2014;15:R46. https://doi.org/10.1186/gb-2014-15-3-r46
2. Chklovski A et al. CheckM2: a rapid, scalable and accurate tool for assessing microbial genome quality using machine learning. *Nat Methods* 2023;20:1203–1212. https://doi.org/10.1038/s41592-023-01940-w
